# Breast cancer patient characterisation and visualisation using deep learning and fisher information networks

**DOI:** 10.1038/s41598-022-17894-6

**Published:** 2022-08-17

**Authors:** Sandra Ortega-Martorell, Patrick Riley, Ivan Olier, Renata G. Raidou, Raul Casana-Eslava, Marc Rea, Li Shen, Paulo J. G. Lisboa, Carlo Palmieri

**Affiliations:** 1 School of Computer Science and Mathematics, Liverpool John Moores University, James Parsons Building, Byrom Street, Liverpool, L3 3AF UK; 2 Institute of Visual Computing & Human-Centred Technology, TU Wien, Vienna, Austria; 3 Department of Electronic Engineering, University of Valencia, Valencia, Spain; 4 The Clatterbridge Cancer Centre, NHS Foundation Trust, Liverpool, UK; 5 Department of Neuroscience, Icahn School of Medicine at Mount Sinai, New York, USA; 6 Institute of Systems, Molecular and Integrative Biology, Molecular and Clinical Cancer Medicine, University of Liverpool, Liverpool, UK

**Keywords:** Cancer, Breast cancer, Computer science

## Abstract

Breast cancer is the most commonly diagnosed female malignancy globally, with better survival rates if diagnosed early. Mammography is the gold standard in screening programmes for breast cancer, but despite technological advances, high error rates are still reported. Machine learning techniques, and in particular deep learning (DL), have been successfully used for breast cancer detection and classification. However, the added complexity that makes DL models so successful reduces their ability to explain which features are relevant to the model, or whether the model is biased. The main aim of this study is to propose a novel visualisation to help characterise breast cancer patients using Fisher Information Networks on features extracted from mammograms using a DL model. In the proposed visualisation, patients are mapped out according to their similarities and can be used to study new patients as a ‘patient-like-me’ approach. When applied to the CBIS-DDSM dataset, it was shown that it is a competitive methodology that can (i) facilitate the analysis and decision-making process in breast cancer diagnosis with the assistance of the FIN visualisations and ‘patient-like-me’ analysis, and (ii) help improve diagnostic accuracy and reduce overdiagnosis by identifying the most likely diagnosis based on clinical similarities with neighbouring patients.

## Introduction

Breast cancer is the most commonly diagnosed female malignancy globally with over two million women diagnosed annually with 685,000 deaths^1^. Outcomes from breast cancer are linked to the stage at diagnosis, with early diagnosis associated with better survival rates^2^. The principle underpinning screening for breast cancer is that detecting the disease at an early stage and the institution of effective management, treatment and follow-up earlier in the disease process will improve the patients’ outcomes. The NHS breast cancer screening programme commenced in 1988 following the Forrest report^3^ and is based on three-yearly mammography to detect changes in the breast tissue that may indicate the presence of cancer. The potential benefits and the harms of mammography, particularly overdiagnosis, have been extensively discussed, most recently in the Marmot report^4^.

Magnetic resonance imaging (MRI) can also be used to detect breast cancer and it is a more comprehensive test used for screening, recommended especially for high-risk patients. However, a breast MRI has a much higher false-positive rate than mammography as they highlight both cancerous and benign tumours with little difference in the characteristics of both, triggering more tests and biopsies. Breast MRIs are also costlier than mammograms and are less available. The UK’s National Institute for Health and Care Excellence does have a stratified surveillance programme for patients in different risk groups (including breast density) on who should be offered a mammogram, an MRI or both^5^. Having a mechanism helping to elucidate further insights from mammograms to reduce overdiagnosis as well as missing cancers would be highly beneficial, especially before the need to request a biopsy.

The application of Machine learning (ML) in healthcare, and particularly breast cancer classification, has proven to be successful. Various studies in the problem area^6–11^ have shown the suitability of ML to discriminate between tumours and classify effectively in a manner of approaches. More recently, models developed^12–14^ using deep learning (DL), a subset of ML algorithms, have shown the capability to attain strong predictive outcome measures, although at the expense of sacrificing the ability to explain how the model arrived at its conclusions or was making its decisions.

Convolutional Neural Networks (CNN) are one of the most representative DL algorithms, extensively used in many aspects of medical image analysis, and allowing for great progress in this area in recent years^15^. CNNs consist of multiple layers of artificial neurons, each of them extracting features that are passed to the next layer for more complex representations or features, which eventually will enable the recognition of objects, etc. This work studies features that are extracted from the deepest layer (before the dense layer) of a CNN model, called convolutional features. This is done to take advantage of the CNN model's effective representation of the information, which has led to highly accurate prediction results reported in this area^14,16–20^.

As ML models become more complex, such as CNN models, the level of explainability reduces, i.e. it becomes more difficult to understand what variables/features the model is taking into account to make a prediction, or whether the model is biased. Common approaches such as Saliency Maps and GradCAM have been used to help understand which parts of an image a CNN model found of interest to make a prediction. However, recent studies^21,22^ have reported that the evaluation of these techniques in carefully-designed user studies has lagged behind technique development, in some cases providing explanations that users cannot understand or find too complex.

Understanding how a model makes predictions is especially relevant in healthcare applications, and it is a major driver for clinical take-up, enabling clinicians to accept and trust these technologies so that they become part of routine clinical practice^23^. Hence our focus in this work is to propose a novel method that provides that explanation, not in the form of saliency features, but instead as a smart representation of the complete dataset in a low-dimensional space, that can provide intelligence about a specific data point based on its neighbouring data points. This is what we call a ‘patient-like-me’ approach.

The proposed patient-like-me approach could help not only to identify the most likely patient’s diagnosis but even potentially the patient’s prognosis based on the clinical similarities with some other patients. It could also inform the possible course of treatment, as it would allow checking the kind of treatments or therapies that were (and were not) successful for those nearby cases, playing a role in the decision-making process of a clinician. Lastly, it could help reduce overdiagnosis as well as missing cancers.

To achieve this, our method involves the use of the extracted convolutional features to form new input data to train a Multi-Layer Perceptron (MLP). This is then followed by the creation of a Fisher Information (FI)^24–26^ network using probability density estimates over the classes involved in the study, used to inform a learning metric (FI metric) in a latent variable space. The FI metric measures dissimilarity for small changes between the data points according to their degree of relevance with respect to class membership. The resulting visualisation of the latent space can be used to project the current and future observations, enabling the analysis of individual cases in the context of all the other cases. Used in a medical application, this can be used to provide a representation for all patients and to enable the study of individual ones, which enhances the clinical understanding, confidence, and take-up.

This global view of the data derived by the FI metric displays a meaningful structure that implicitly informs about underlying class probabilities. The FI network framework, which has been successfully used in previous studies^27–30^, can be used not only to create a meaningful neighbourhood mapping to visualise data but also to create an interpretable retrieval-based classifier, as connection weights contain accurate information about data points' similarity. For the proposed methodology, an FI network is constructed using probability density estimates, which are calculated across five classes from a model created by Shen et al.^16^, to produce a visualisation of the latent space of breast cancer patients using convolutional features extracted from mammography images. Shen’s mammogram patch model discriminates between five classes—four lesions and one image background—and is used to inform a transfer learning model on full images over two classes detecting the presence of cancer or not in a full mammogram.

We expect that a visualisation of the latent space obtained with the FI network will help elucidate the underlying data structure, with the ultimate aim of assisting the diagnosis of new patients. The latter can be achieved by projecting new unseen instances/observations into this latent space, given that a huge amount of information can be learnt from the closest neighbours, which would be potentially relevant to those new patients. The proposed approach will be compared against a new state-of-the-art method called uniform manifold approximation and projection (UMAP)^31^, which has been reported^32^ to be more efficient than *t*-distributed stochastic neighbourhood embedding (t-SNE)^33^ in terms of computation time and data representation.

For the analysis and evaluation of the proposed method, we used an existing, publicly available dataset of mammograms, namely the Curated Breast Imaging Subset of the Digital Database for Screening Mammography (CBIS-DDSM)^34^. This dataset has been used previously for classification tasks^14,16–18,35–38^, for which the primary focus is to attain strong predictive performance, achieved using DL.

The aims of this study are, from the methodological point of view, to:

1. Propose a novel visualisation of a meaningful neighbourhood mapping containing accurate information about data points' similarities that can provide intelligence about neighbouring data points.
2. Ensure that the resulting visualisation can be used for training and independent test cases, enabling the analysis of future observations in the context of all the other cases.

And from the clinical point of view, to:

1. Apply this methodology to breast cancer data and assess whether the visualisation of all patients can assist the study of individual ones (‘patient-like-me’ approach).
2. Facilitate the analysis and decision-making process in breast cancer diagnosis with the assistance of this novel visualisation and help improve diagnostic accuracy and reduce overdiagnosis.

## Methods

### Description of the dataset

In this study, we used a publicly available dataset, the Curated Breast Imaging Subset of the Digital Database for Screening Mammography (CBIS-DDSM)^34^, which is a standardised version of the Digital Database for Screening Mammography (DDSM)^39^. The DDSM is a rich mammography database, containing over 2000 scanned film mammography studies with verified pathology information from 1246 women. The CBIS-DDSM includes a subset of the DDSM data selected and curated by trained mammographers. Within the dataset, updated regions of interest (ROI) from the images have been provided and the pathologic diagnosis is also included.

For this dataset, both craniocaudal (CC) and mediolateral oblique (MLO) views are available for most of the exams, which are the views usually used for routine screening mammography. It also contains associated metadata for each lesion, including the BI-RADS assessment (BI-RADS stands for Breast Imaging Reporting and Data System). The latter is traditionally used to assess lesions found in different mammography images into categories, depending on their severity. BI-RADS score ranges from 0 to 6, however in this dataset, only scores from 0 to 5 are available. This dataset contains both breast masses and calcifications and holds a pathology label for each case, which is either *benign* or *malignant* with verified pathology information. We used the same split separation of training and test cases followed by Shen et al.^16^, which used an 85–15 split at the patient level, stratified to maintain the same proportion of cases per class in the training and test sets.

### Visualising the latent space of breast cancer patients—proposed methodology

**Figure 1:**
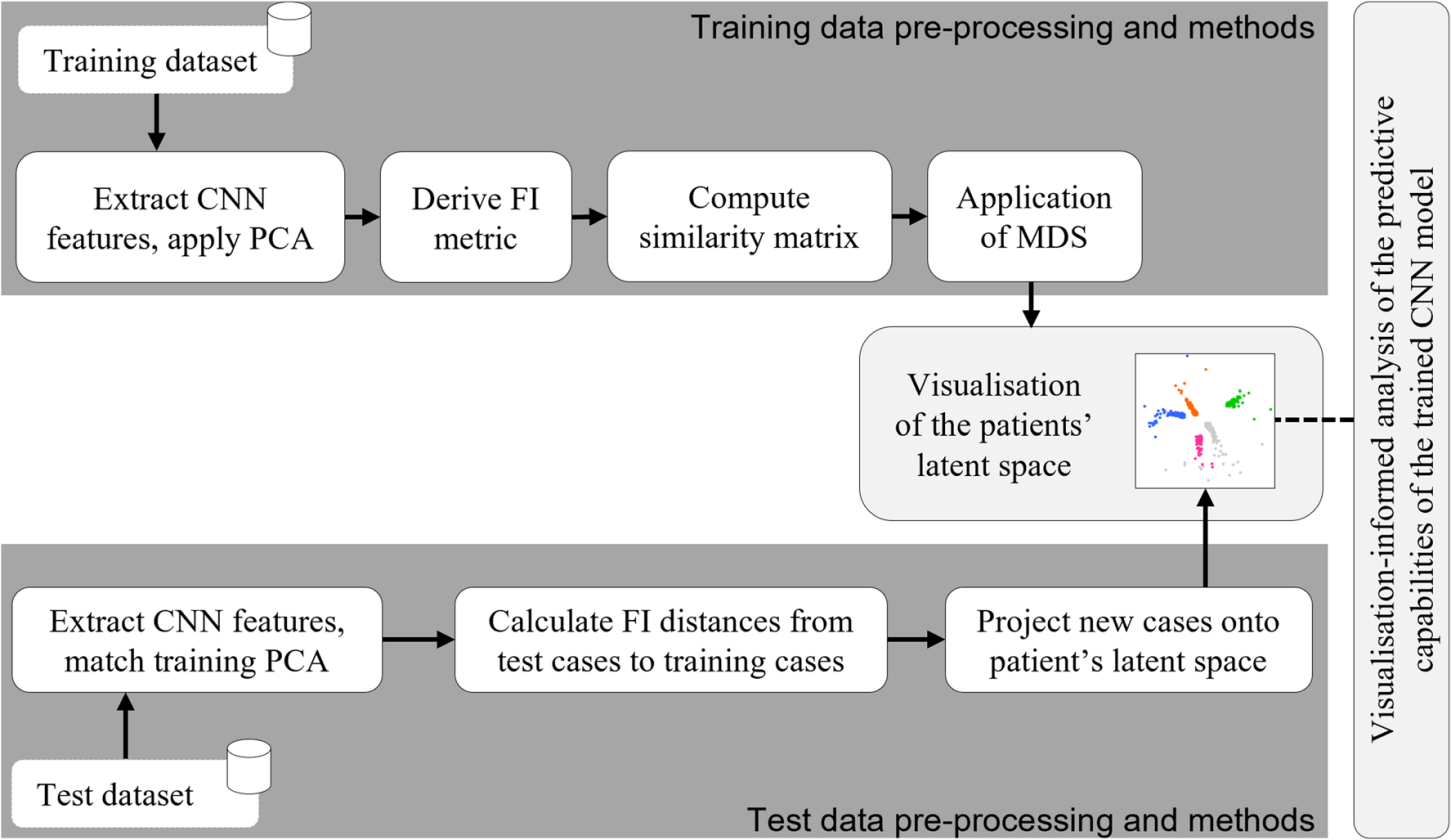
Proposed methodology. Utilising the FIN to create a visualisation of the latent space of cancer patients, exploiting the predictive capabilities of a deep learning model.

The main objective of this study is to create a visualisation of the latent space of breast cancer patients that represents the variability that can be found in the dataset, from which we gain insights on how different patients can be related, leading to the development of a ‘patient-like-me’ approach. To achieve this, we propose a methodology illustrated in Fig. 1.

#### Extracting CNN features and developing MLP model

To study a CNN classifier as proposed, this work required a strongly performing deep learning model. The Resnet50-based model^40^ is a 5-class patch classifier, trained on patches of mammograms around the lesion. Using the CBIS-DDSM dataset, the five classes are: calcification malignant, calcification benign, mass malignant, mass benign and (image) background. It is used later in Shen’s work to develop a new classifier through transfer learning to inform a 2-class full image model, discriminating between the presence or not of cancer. A description of any data pre-processing, how the patches were attained and the curation of the five classes is described in Shen et al.^16^.

This method can be computationally expensive and therefore to reduce the extracted 2,048 extracted CNN features, PCA has been used for dimensionality reduction. Out of the 2048 CNN features, 1,363 principal components are kept after applying PCA, representing nearly 67% of the original number of features, and capturing 90% of the variance. This CNN is used as a feature extractor in this work—the outputs of the penultimate layer are extracted and used as the dataset for this work.

Using the CNN features as the input dataset, an MLP was developed to discriminate between the five classes. The MLP was initialised with random weights, one hidden layer of 30 nodes and an output layer, a learning rate of 0.01 and a momentum of 0.9. Weight decay regularisation was implemented at a rate of 0.2. As a five-class classifier, the activation function was soft-max.

#### Creating the latent space of breast cancer patients

Firstly, the FI metric is derived using the probability densities of the classes estimated with the MLP and is then used to calculate pairwise distances. The FI is usually defined in terms of the joint distribution of data values, *x*, and a parameter set, $\theta$, by integrating over the data to obtain a function of a given parameter. In Ruiz et al.^26^ this is reversed and the posterior probability of class membership $p\left(c|x\right)$, estimated with an MLP, forms the basis to calculate the FI by summing over both classes, so that it is a function of a given data point, *x*, as follows (see Eq. 1):

1 $$FI\left( x \right) = \mathop \sum \limits_{c} \left( {\nabla_{{\text{x}}} {\text{log}} p\left( {c|x} \right)} \right)\left( {\nabla_{{\text{x}}} {\text{log}} p\left( {c|x} \right)} \right)^{T} p\left( {c|x} \right) = - \mathop \sum \limits_{c} \nabla_{{\text{x}}}^{2} {\text{log}} p\left( {c|x} \right)p\left( {c|x} \right)$$

where ${\nabla }_{x}$ is the gradient with respect to $x$. The theoretical justification for this equation is explained in Appendix A of Ruiz et al.^26^. It has the standard form of the FI, expressed in two equivalent ways.

It is well known that the FI matrix is a metric which therefore generates a Riemannian space over the range of the input data. This forms the basis for using the posterior probability to map the data into a structure based on similarity, which is measured locally by the quadratic differential form in Eq. 2:

2 $$d\left( {x,x + \Delta x} \right)^{2} = \Delta x^{T} FI\left( x \right)\Delta x$$

The distance between a pair of data points is ideally measured along a geodesic, but this is computationally very expensive. It is shown in Ruiz et al.^26^ that a good approximation is obtained in a computationally efficient manner with the Floyd-Warshall algorithm^41,42^. This involves calculating the shortest path between distant points by hopping from each data point to a nearest neighbour, estimating the geodesic distance between neighbouring points by integrating (2) over a straight-line path, for which an analytical expression is given in Ruiz et al.^26^ (this is Eq. 11 in the referred study^26^).

Using the distance matrix, the similarity matrix is then calculated through the use of a Gaussian radial kernel, which produces an adjacency matrix that defines the network structure (see Eq. 3).

3 $$A = {\text{exp}}\left( { - \frac{{\Delta x^{2} }}{{\sigma_{G}^{2} }}} \right)$$

where the Gaussian kernel width *σ*_*G*_ is a natural length which was taken to be the average pairwise geodesic distance between points belonging to the same predicted label.

Multidimensional scaling is utilised to represent the data in a low-dimensional Euclidean space, by matching as closely as possible the distance between respective cases in higher dimensional Riemannian space. Since this is now a projective space, the possibility of using signal-processing techniques is enabled, still retaining the structural representation identified by the FI matrix. The obtained visualisation of the patients’ latent space facilitates the ‘patient-like-me’ approach.

#### Application to new, unseen patients

To satisfy the ‘patient-like-me’ objective of this paper, test cases are projected onto the latent space of trained observations. It is expected that, without informing the model of the pathology of the cases in the test set, some characteristics of these cases will be revealed as they would fall in the proximity of similar cases. To achieve this, the test data go through similar steps in the methodology as the training data, which is also illustrated in Fig. 1. Firstly, CNN features were extracted from the test set and PCA was applied, performing the same data transformations followed for the training set. Then, the trained MLP model was applied to the test data to estimate the class probabilities of each observation, which were then used as part of the calculations of the FI distances from each test case to each of the training cases. Based on this, and mapping the test cases back to the Euclidean space, each of them is then projected on the pre-constructed latent space (created using the training data), where it can then be visualised and analysed against its neighbouring cases.

## Results

### Model results

Although the classification results are not the focus of this work, they have been included to structure the discussion of utilising the visualisation to assess the capabilities of the patch model in Shen et al.^16^. Table 1 shows the confusion matrix for the independent test set. The overall accuracy in the test set is 72%.

**Table 1:** Confusion matrix of the CNN classifier for the test set.

Test set confusion matrix (72% overall accuracy)		Predicted				
		Background	Calcification Benign	Calcification Malignant	Mass Benign	Mass Malignant
Actual	Background	0.90	0.04	0.02	0.03	0.02
	Calcification Benign	0.30	0.57	0.09	0.01	0.03
	Calcification Malignant	0.13	0.26	0.46	0.03	0.11
	Mass Benign	0.25	0.02	0.01	0.57	0.15
	Mass Malignant	0.10	0.01	0.10	0.12	0.66

The rows are normalised (each sums to one) to show the correctly classified proportion in each class, namely: Background, Calcification Benign, Calcification Malignant, Mass Benign and Mass Malignant.

### Exploration using Principal Component Analysis (PCA)

**Figure 2:**
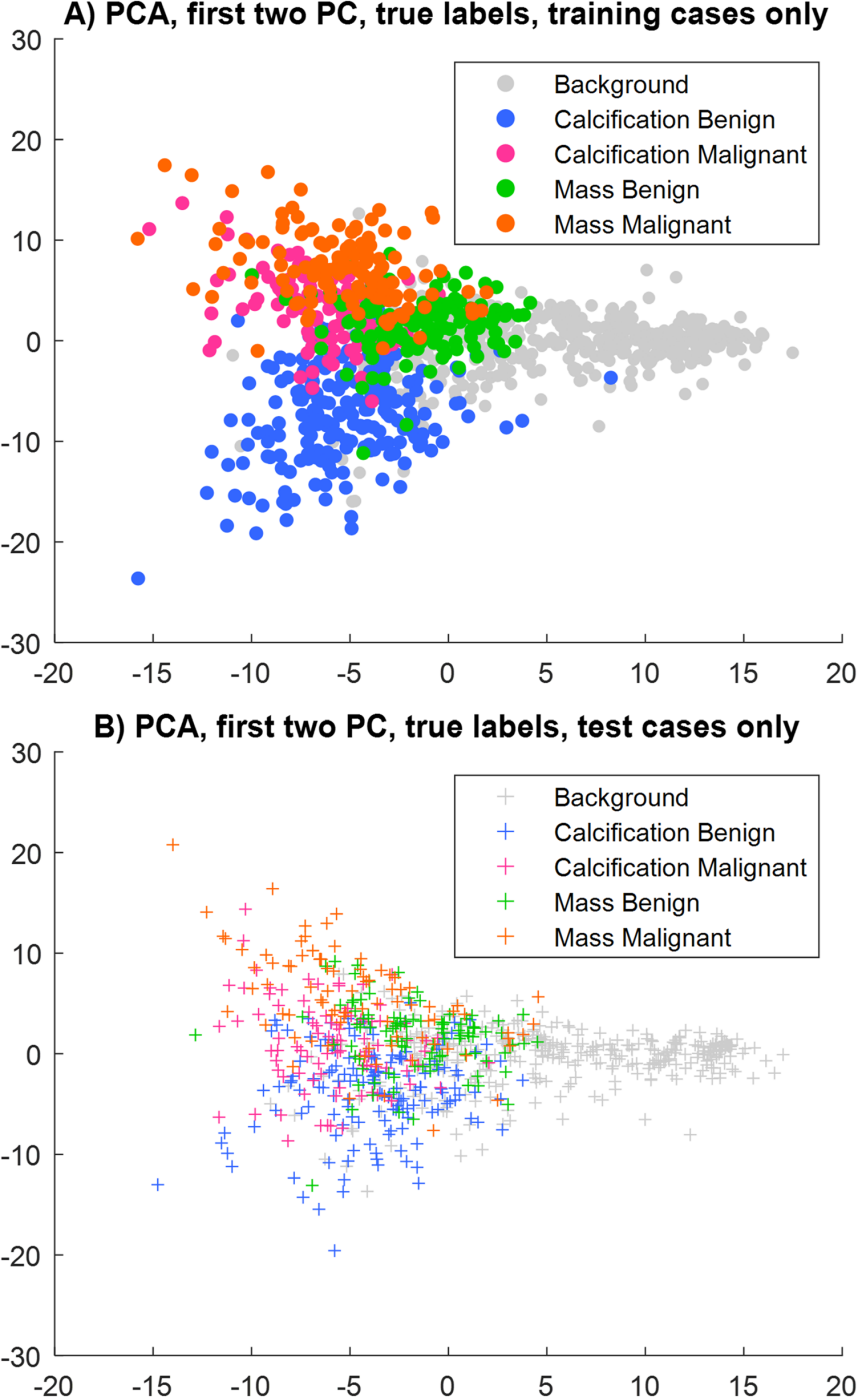
Visualisation using Principal Component Analysis. Convolutional features extracted are projected on the first two principal components (PC). Data points represent patient mammograms, which are colour-coded by their assigned label. (**A**) shows only the training data; (**B**) only the test data.

PCA analysis on the extracted features has been used to visualise the separation of the classes without applying the FIN process, as well as for dimensionality reduction before the process. Figure 2 shows the first two principal components (PC) when applying PCA on the convolutional features, where the data points represent the patient’s mammograms colour-coded by the four true (originally assigned) labels and the background area. Figure 2A visualises the training data and Fig. 2B visualises both the training and test data, both before applying the FIN methodology. Although some grouping can be seen across both training and test, the mixing of the classes is apparent. All classes overlap in the centre of the visualisation, and both malignant classes are deeply mixed. There is some improvement with the benign classes however mixing still occurs.

### Visualisation using Uniform Manifold Approximation and Projection (UMAP)

**Figure 3:**
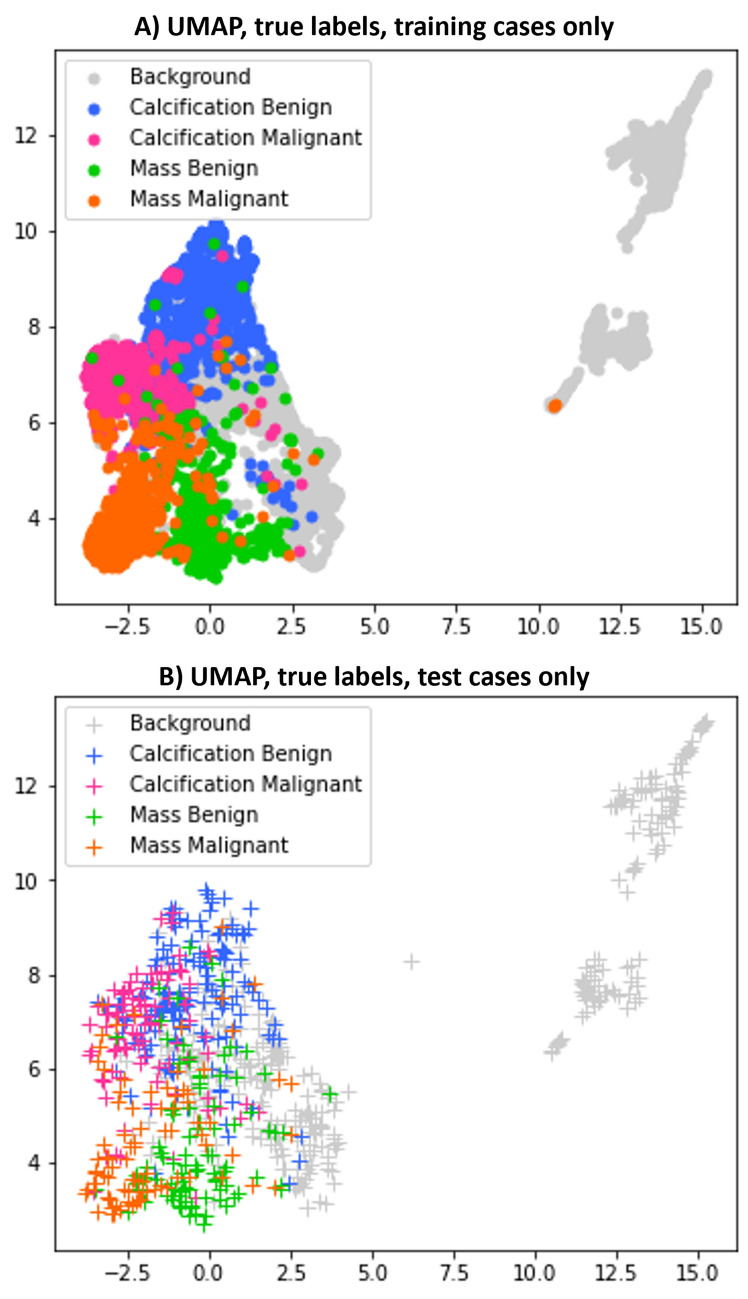
Visualisation using supervised UMAP. Supervised UMAP is applied to the set of extracted convolutional features. As in Fig. 2, data points represent patient mammograms, which are colour-coded by their assigned label. (**A**) shows only the training data; (**B**) only the test data.

UMAP^31^ is a non-linear dimension reduction algorithm that can be used to learn an embedding of the data for an alternative ‘patient-like-me’ approach. It is a versatile method that can be used in unsupervised and supervised ways. In this study, both versions (unsupervised and supervised) of UMAP were applied to the set of extracted convolutional features. As expected, the supervised UMAP performed better than the unsupervised, and several tests were performed varying different parameters, e.g. the number of neighbours. Figure 3 shows the results obtained with supervised UMAP for training and test sets when using 15 neighbours and 42 random states. The groupings in Fig. 3 seem much more clear than those obtained with PCA (Fig. 2), although still there is mixing, especially notorious in the test set. It is also interesting to see how a good proportion of the class that represents image background was well separated from the rest of the lesions.

### FIN visualisations of the training cases

**Figure 4:**
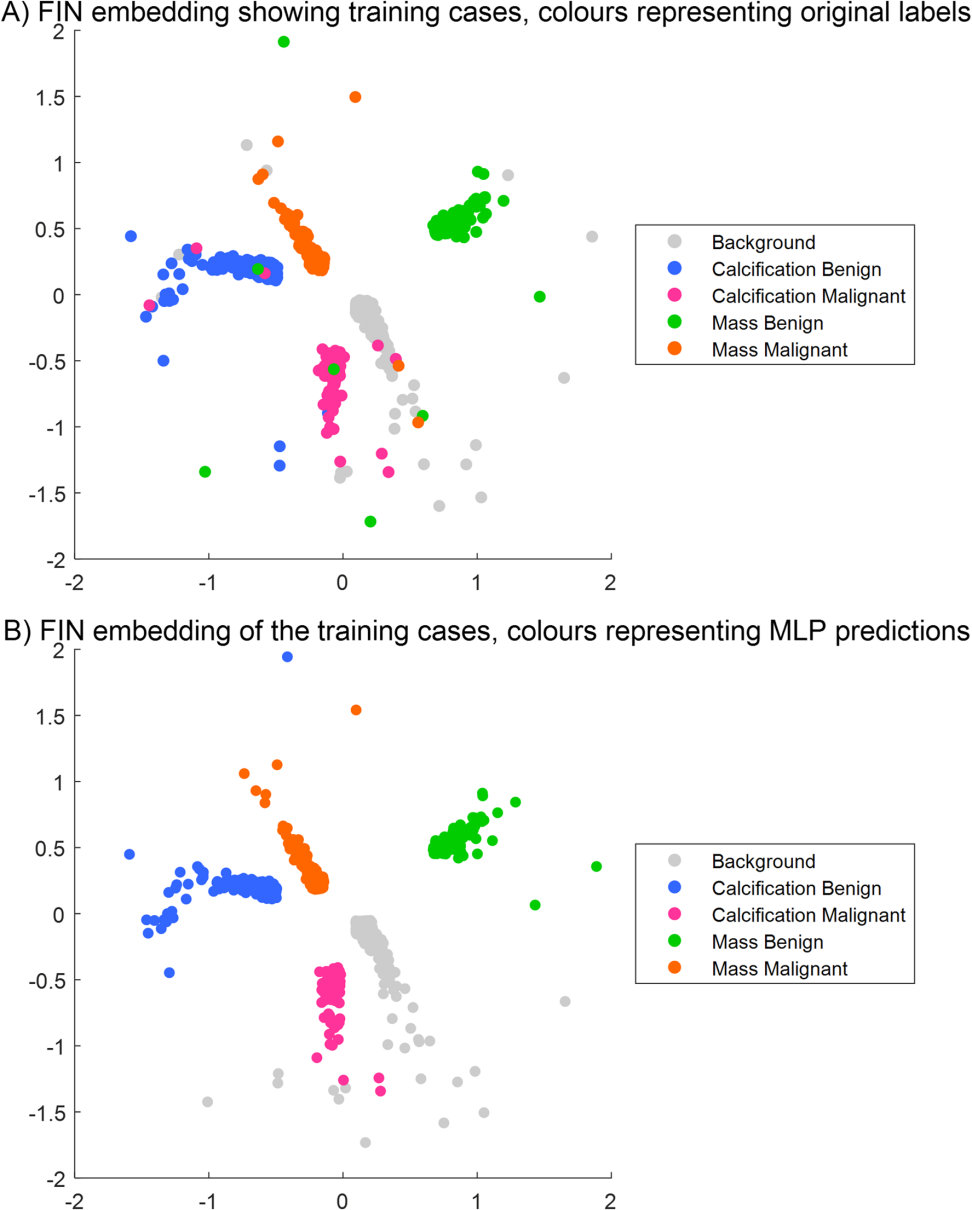
Visualisation of the training cases in the latent space of the FIN embedding. Cases/patient mammograms are represented by points: (**A**) colours representing the true, original labels; (**B**) colours representing the MLP prediction labels.

Figure 4A shows the visualisation of the latent space obtained with our proposed approach using the FIN methodology on the convolutional features derived from the DL model of the five-class classifier. Similar to Figs. 2 and 3, the data points also represent the patient’s mammograms colour-coded by the four true (originally assigned) labels and the background area. However, as opposed to Figs. 2 and 3, only very limited mixing can be seen.

Figure 4B shows the visualisation but of how the model predicted the training cases. This is an expected result and gives a view of how the classification process of the CNN model works. In Fig. 4B the classes are very well separated with most of the mixing occurring between the background and the calcification malignant classes. This figure can be used to assess how well the FIN representation reflects the MLP classifier. Through comparison of the visualisation with the true labels applied and the MLP predictions applied, the FIN representation looks to be suitable. The MLP structure is well reflected in this study and can be seen as a reliable mapping of the training cases, for which test cases will be projected onto at a later stage.

### Projecting the test cases onto the trained embedding

**Figure 5:**
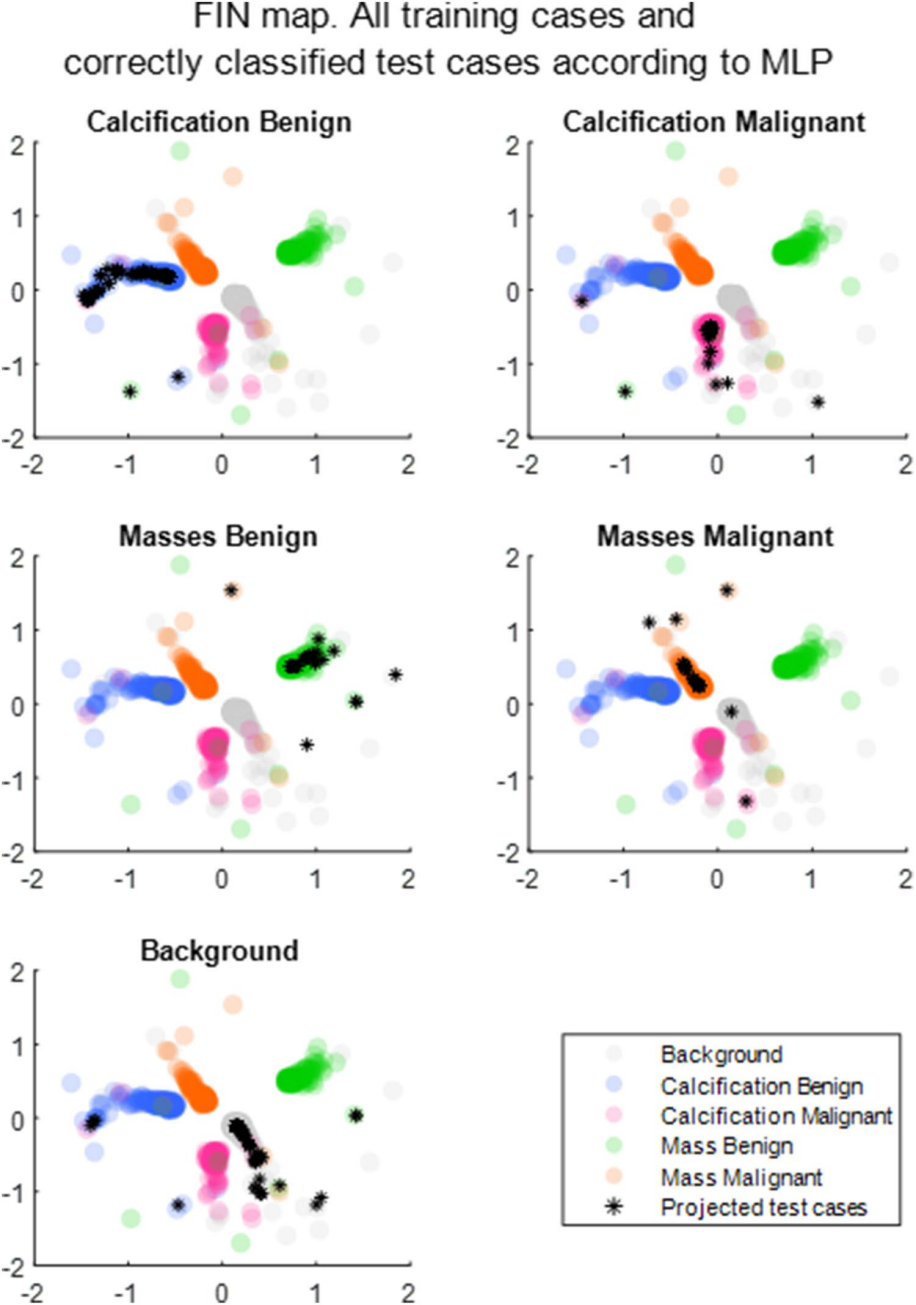
FIN map: all training cases and correctly classified test cases according to the MLP. Correctly classified test cases (black stars) projected into the trained FIN embedding, where every case is represented by a point—transparency was added mainly to allow for overlapping cases to be seen, and as a result, areas with the majority of cases in one class will display a more solid colour.

Figure 5 shows the correctly classified test cases of each class highlighted as a black marker on top of the more transparent training cases. These are the cases that have been classified correctly. For the test subset of the data, the background class contains the most correctly classified cases, with an accuracy of 89.6%. It is possible to see some of these correctly classified cases slightly spread out although these appear to be concentrated around the benign classes. The next best performing is the mass malignant class, with 66.3% of those test cases correctly classified.

The visualisation in Fig. 5 shows that the correctly classified cases are projected within the well-defined class groups. However, not all cases are correctly classified. A more detailed analysis of some of these incorrectly classified cases has been conducted, to assess the model and better understand the reasons behind the misclassifications.

### Detailed analysis of individual cases

Similar to the previous section, it is proposed that these trained embeddings can be utilised as a part of a decision support system for clinicians and radiologists in the diagnostic process of a patient’s treatment care plan, which could help reduce the need for a pathological label in several cases. Through the extraction of the convolutional features, using the same pre-trained model can lead to the new case being projected onto the embedding. This can provide new insight into the case as it can be studied in the context of the neighbouring cases in the embedding, i.e. a ‘patient like me’ analysis.

**Figure 6:**
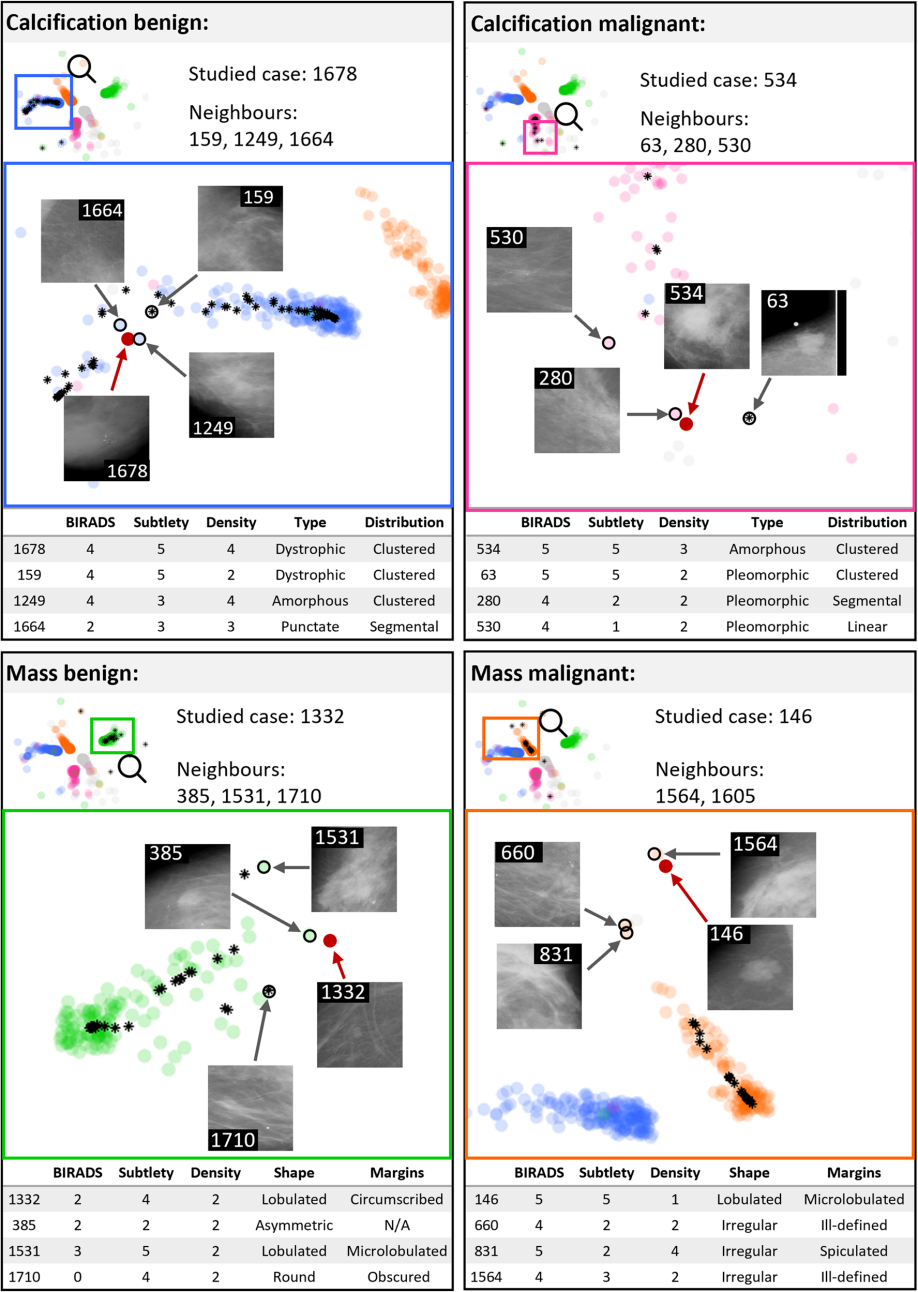
‘Patient like me' analysis of four selected cases that were correctly classified. Top left: patient 1678 (calcification benign); top right: patient 534 (calcification malignant); bottom left: patient 1332 (mass benign); and bottom right: patient 146 (mass malignant).. Cases are represented by points with added transparency as in Fig. 5. Black stars are the correctly classified test cases in each of the four groups, and the selected cases are represented with solid red circles. Neighbouring cases were also selected and visualised (empty black circles) for each of the four previous cases.

Figure 6 shows a ‘patient like me’ analysis of four correctly classified cases. Additional metadata, unseen by the classifier and also unseen by the proposed FIN approach, has been included to assess these individual cases and understand the outcome of the classification to validate the proposed approach. Figure 6 highlights the location of where these four selected test cases were projected in the trained embedding, along with a selection of neighbouring cases, and their corresponding ROI patches. All malignant cases shown here have high BI-RADS scores of 4 and 5, suggesting concern and high suspicion of malignancy, respectively. For the calcification malignant cases in Fig. 6, they are all of the type amorphous or pleomorphic. This follows clinical literature as these are more likely to be malignant lesions^43^. Similarly, for the malignant mass cases, these are all of irregular or lobulated shapes which are more likely to be malignant.

**Figure 7:**
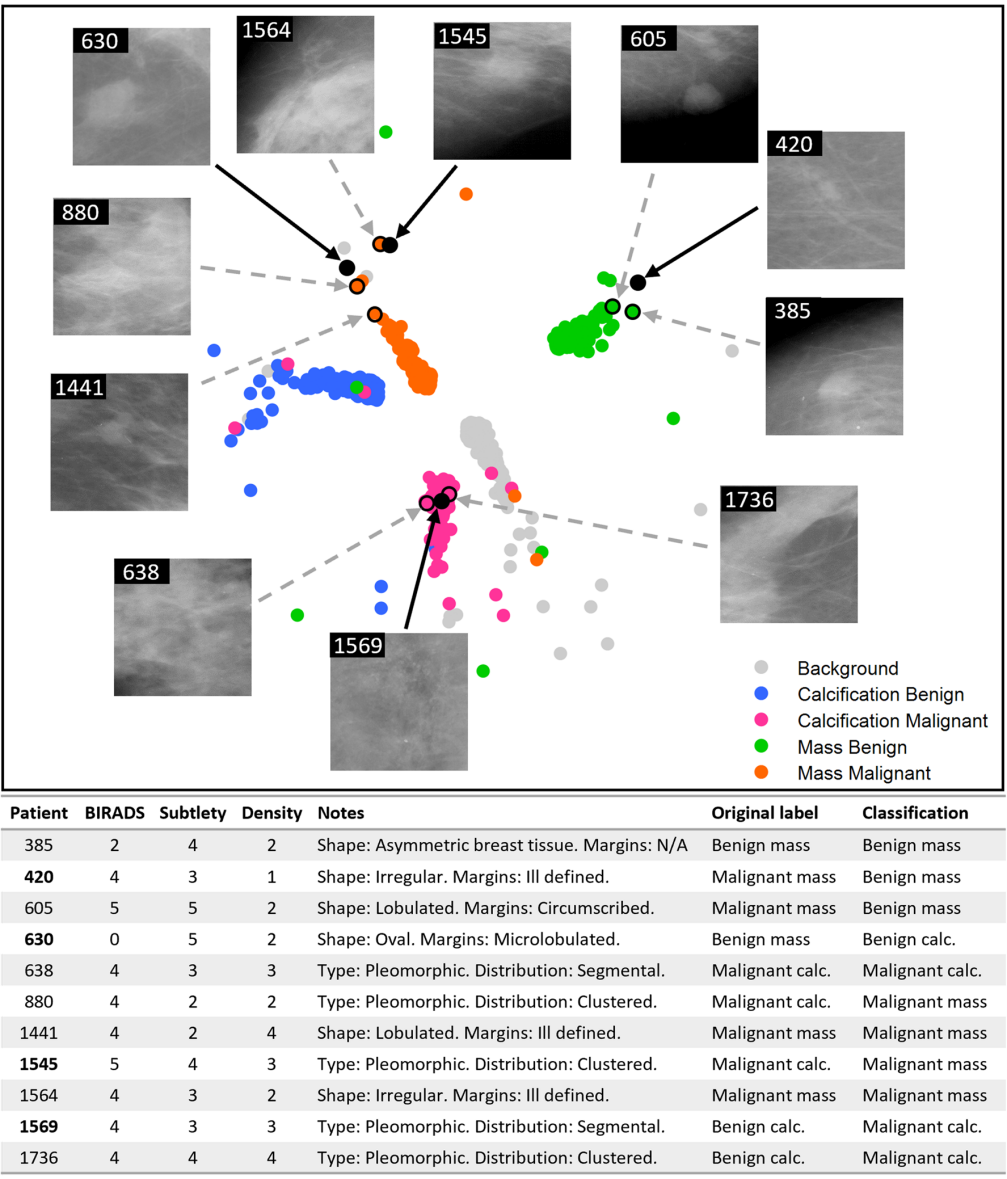
‘Patient like me' analysis for four misclassified cases. Patient 1569 is a misclassified benign calcification (classed as malignant calcification), patient 1545 is a misclassified malignant calcification (classed as malignant mass), patient 630 is a misclassified benign mass (classed as benign calcification), and patient 420 is a misclassified malignant mass (classed as benign mass). The selected cases are represented with black solid circles.

Figure 7 presents a similar ‘patient like me’ analysis for four cases that were misclassified. Importantly for these cases, the metadata can provide insight into why the cases were misclassified. For example, for both case 1569—a calcification benign case incorrectly classified as calcification malignant—and case 420—a mass malignant case incorrectly classified as mass benign—the associated metadata shows that the BI-RADS score assigned is 4 (BI-RADS was not a feature in the classification, and is denoted from studying a mammogram alone, pre-pathology). This score of 4 is defined as ‘suspicious of malignancy’, with a score of 3 or 5 leaning towards benign or malignant, respectively. This could support the fact that the cases were misclassified.

## Discussion

The focus of this work is to propose an ML methodology able to (1) facilitate the analysis and decision-making process in breast cancer diagnosis, which is key for the reduction in mortality from breast cancer; (2) help improve the diagnostic accuracy, to avoid both, false negatives (missed cancers) and false positives (which involves a psychological impact and recall for further tests)^44^; and (3) help reduce overdiagnosis, one of the major harms reported of the well-established breast cancer screening programmes^4^. To achieve this, the proposed methodology was applied in this study to mammography data, to visualise all patients in a single latent space from where comparisons and associations between patients can be made, aiding diagnosis and further clinical investigations.

To illustrate how it works, the proposed methodology started from a pre-trained model available in the literature^16^, which provides a 72% classification accuracy on test data. This pre-trained model is a 5-class patch classifier implemented and used in Shen et al.^16^ as an intermediate step to determine whether there is cancer or not in a mammogram. However, this methodology is not restricted to the specific pre-trained model referenced—it can also be applied to any other available or newly trained model.

The trained embeddings shown in Fig. 4 provide clear indication of the quality and usefulness of the separation using the FIN framework, showing its ability to greatly improve the five class separation as compared with the ones obtained on the same data with PCA (Fig. 2) and the supervised UMAP (Fig. 3). Figure 4 also shows that this methodology can accurately represent the model trained using the CNN features derived from the mammograms, and produce a visualisation of a latent space where a large set of patients are mapped out according to their similarities. The major benefit of the latter is that not only further knowledge and understanding can be generated, but also that new patients can be added or projected onto this embedding, which would provide intelligence about them based on their neighbouring patients in the visualisation.

As per the patient-like-me analysis, we have shown that it is possible to study a new case (that has been unseen to the classifier) in the context of those around it, to gain extra insight into it. For example, the correctly classified cases (as shown in Fig. 6) can be used as examples of correctly labelled and classified patients. Studying the associated training cases within the embedding can strengthen the confidence in the findings arising during the investigation of new patients.

The proposed method has not been correct in the classification of 28% of the test cases since the proposed approach relies on the quality of the initial CNN model to produce the embedding. However, it is worth noting that several of those misclassified cases (as shown in Fig. 7) had BI-RADS scores that would indicate suspicion of malignancy, the borderline score in this classification, which may justify the decision made by the classifier based on the features extracted automatically from the mammograms. The selection of the model is justified through its strong predictive capabilities originally, and its use is for the features of the publicly available dataset and model.

It is intended that the FIN methodology proposed can be used to provide useful and insightful visualisations to clinicians and radiologists, for the use of this extra information to assist in a ‘patient like me’ approach to attain further insights into the workings of the classifier and visualisations. To improve this further, future work could consider the use of active learning^45^ to continuously improve the embedding. Before any pathology is taken, a case can be projected onto the FIN embedding and studied in the context of their neighbouring cases. Then, where pathology is taken, this information can be included and the methodology updated to improve the model, leading to a better ‘patient like me’ approach for future patients.

One of the endeavours of this study was to exploit the predictive capabilities of a deep learning model. The FIN methodology has helped to further the understanding of how the CNN classification process works in this study. The Fisher Information metric has provided a robust mechanism to maximise the separation between the different classes, whilst retaining the cohesion of the group of cases with similar characteristics and has allowed for a visual representation of a pre-trained model which exploits the predictive capabilities of an image classification task using CNN. Associated information using clinical literature is able to append reasoning to the classification, such as information on the shape or type of lesion and the BI-RADS scores. This assists with the link between the human clinician and the machine learning classifier.

The use of the FIN methodology shows that this process can be applied to represent clinical data, classified through a CNN model which is very powerful but traditionally difficult to interpret. A Fisher-informed approach can add insight into the predictive capabilities and as described, can assist in the breast cancer diagnostic process.

In summary, in this study we propose a novel visualisation using FIN containing accurate information about data points' similarities that can provide intelligence about neighbouring data points. This was successfully applied to training and independent test cases and enabled the analysis of breast cancer data using a ‘patient-like-me’ approach. We showed how this visualisation could be used in a clinical setting to better understand the characteristics of a patient and facilitate the analysis and decision-making process in breast cancer diagnosis, which may help improve diagnostic accuracy and reduce overdiagnosis. A limitation of the proposed method is that the calculation of the FI distances when creating the embedding may be slow depending on the number of data points and the sizes of the images. However, existing implementations can be used in a high-performance computing cluster which can reduce the time considerably.

## Data Availability

The dataset analysed in the current study is publicly available in the Cancer Imaging Archive repository, https://wiki.cancerimagingarchive.net/display/Public/CBIS-DDSM.

## Abbreviations

BI-RADS: Breast Imaging Reporting and Data System
CLAHE: Contrast Limited Adaptive Histogram Equalisation
CNN: Convolutional Neural Network
FI: Fisher Information
FIN: Fisher Information Network
MDS: Multidimensional Scaling
ML: Machine Learning
MLP: Multi-Layer Perceptron
ROI: Region of Interest
